# *Ex vivo* Validation of Noninvasive Epicardial and Endocardial Repolarization Mapping

**DOI:** 10.3389/fphys.2021.737609

**Published:** 2021-10-22

**Authors:** Jeanne G. van der Waal, Veronique M. F. Meijborg, Charly N. W. Belterman, Geert J. Streekstra, Thom F. Oostendorp, Ruben Coronel

**Affiliations:** ^1^Department of Experimental and Clinical Cardiology, Amsterdam University Medical Centers, Location Academic Medical Center, Amsterdam, Netherlands; ^2^Department of Biomedical Engineering and Physics, Amsterdam University Medical Centers, Location Academic Medical Center, Amsterdam, Netherlands; ^3^Donders Institute for Brain, Cognition and Behaviour, Radboud University Medical Centre, Nijmegen, Netherlands; ^4^IHU Liryc, Electrophysiology and Heart Modeling Institute, Fondation Bordeaux Université, Pessac-Bordeaux, France

**Keywords:** equivalent dipole layer, electrocardiographic imaging (ECGI), noninvasive imaging, inverse problem of electrocardiography, repolarization heterogeneity

## Abstract

**Background:** The detection and localization of electrophysiological substrates currently involve invasive cardiac mapping. Electrocardiographic imaging (ECGI) using the equivalent dipole layer (EDL) method allows the noninvasive estimation of endocardial and epicardial activation and repolarization times (AT and RT), but the RT validation is limited to *in silico* studies. We aimed to assess the temporal and spatial accuracy of the EDL method in reconstructing the RTs from the surface ECG under physiological circumstances and situations with artificially induced increased repolarization heterogeneity.

**Methods:** In four Langendorff-perfused pig hearts, we simultaneously recorded unipolar electrograms from plunge needles and pseudo-ECGs from a volume-conducting container equipped with 61 electrodes. The RTs were computed from the ECGs during atrial and ventricular pacing and compared with those measured from the local unipolar electrograms. Regional RT prolongation (cooling) or shortening (pinacidil) was achieved by selective perfusion of the left anterior descending artery (LAD) region.

**Results:** The differences between the computed and measured RTs were 19.0 ± 17.8 and 18.6 ± 13.7 ms for atrial and ventricular paced beats, respectively. The region of artificially delayed or shortened repolarization was correctly identified, with minimum/maximum RT roughly in the center of the region in three hearts. In one heart, the reconstructed region was shifted by ~2.5 cm. The total absolute difference between the measured and calculated RTs for all analyzed patterns in selectively perfused hearts (*n* = 5) was 39.6 ± 27.1 ms.

**Conclusion:** The noninvasive ECG repolarization imaging using the EDL method of atrial and ventricular paced beats allows adequate quantitative reconstruction of regions of altered repolarization.

## Introduction

Early risk stratification of patients with functional arrhythmogenic substrates may help to reduce mortality caused by arrhythmias. However, the detection and localization of these substrates is not easy and currently involves invasive mapping (Visser et al., 2016). A potential noninvasive tool to overcome this problem is electrocardiographic imaging (ECGI). This technique combines the recording of multiple body surface ECGs with a patient-specific heart-torso geometry to mathematically reconstruct cardiac electrical activity through the solution of the inverse problem. Several inverse calculation methods exist; the most commonly used methods are the epicardial potential (EP) model (Barr et al., 1977; Rudy, 1999; Ramanathan et al., 2004; Cluitmans et al., 2017) and the equivalent dipole layer (EDL) model (Oosterhoff et al., 2016; Janssen et al., 2018; van der Waal et al., 2020). In contrast to the EP method, the EDL method allows simultaneous calculation of both the endocardial and epicardial activation and repolarization times (AT and RT), thereby identifying transmural timing differences that are potentially important for arrhythmogenesis (Antzelevitch et al., 1999; Sabir et al., 2007).

The estimation of ATs using the EDL method is based on the notion that a uniform dipole layer coinciding with the activated portion of the myocardial, endocardial, and epicardial surfaces produces the same potentials at the body surface as the actual activation wavefront that separates the activated and nonactivated myocardium (Geselowitz, 1989). Previous studies using this method have shown good accuracy for reconstructing ATs and origins of premature ventricular complexes (Oosterhoff et al., 2016; Janssen et al., 2018).

The estimation of depolarization sequences is based on an “on-off” scenario (i.e., a tissue is either activated or not). To adapt this assumption to the gradually occurring repolarization phase, Geselowitz has shown that considering a nonuniform dipole layer with a strength that is proportional to the local transmembrane potentials (TMPs) at the myocardial surface allows the inclusion of the complete cardiac cycle (Geselowitz, 1992). However, given the more complex nature of repolarization compared to activation (i.e., less voltage differences, the effect of cardiac motion, and more difficulty in reliably obtaining gold standards), this is more complicated. In a previous *in silico* study, we have shown that this method gives accurate results in reconstructing RTs (van der Waal et al., 2020).

In this *ex vivo* study, we aimed to assess the temporal and spatial accuracy of the EDL model in reconstructing the ventricular RTs from the surface ECG in isolated perfused porcine hearts. In addition, to assess the ability of the method to detect the regions of increased repolarization heterogeneity, these hearts are subjected to interventions that regionally alter repolarization.

## Materials and Methods

All experiments were approved by the institutional review committee for experiments on animals, and animal handling was in accordance with the Dutch Law on Animal Experimentation and the European Directive for the Protection of Vertebrate Animals Used for Experimental and Other Scientific Purposes (European Union Directive 86/609/EEC).

### Experimental Setup

Heart explantation was performed on four male pigs (mean = 62.5, range = 50–80 kg) that had been premedicated, anesthetized, intubated, and artificially ventilated. The animals were heparinized (5,000 IU, Leo Pharma, Amsterdam, The Netherlands), blood was collected, and ventricular fibrillation (VF) was electrically induced to avoid coronary air embolism. After explanation, the hearts were connected to a recirculatory Langendorff perfusion setup with 1:1 blood-Tyrode's mixture kept at 37.5°C (Tyrode's solution (mM): 155.5 Na, 4.7 K, 1.45 Ca, 0.6 Mg, 136.5 Cl, 27.0 HCO_3_, 0.4 PO_4_, 11.1 glucose, pH = 7.35–7.45). By the cannulation of the left anterior descending artery (LAD), selective perfusion of the LAD region and the remaining heart was achieved. After a period of normal perfusion, hearts were selectively perfused with ice-cooled perfusion fluid, creating temperature differences of up to 8°C. In addition, hearts 1 and 2 were LAD-perfused with pinacidil (pinacidil monohydrate P154, 40 μM, Sigma–Aldrich, Zwijndrecht, The Netherlands). Measurements were performed during atrial (cycle length (CL): slow = 600–700 ms, fast = 400–450 ms), right ventricular (RV; CL = 450 ms), and left ventricular (LV; CL = 450 ms) pacing. Intervention measurements were performed during atrial pacing.

### Electrophysiological Recordings

**Figure 1:**
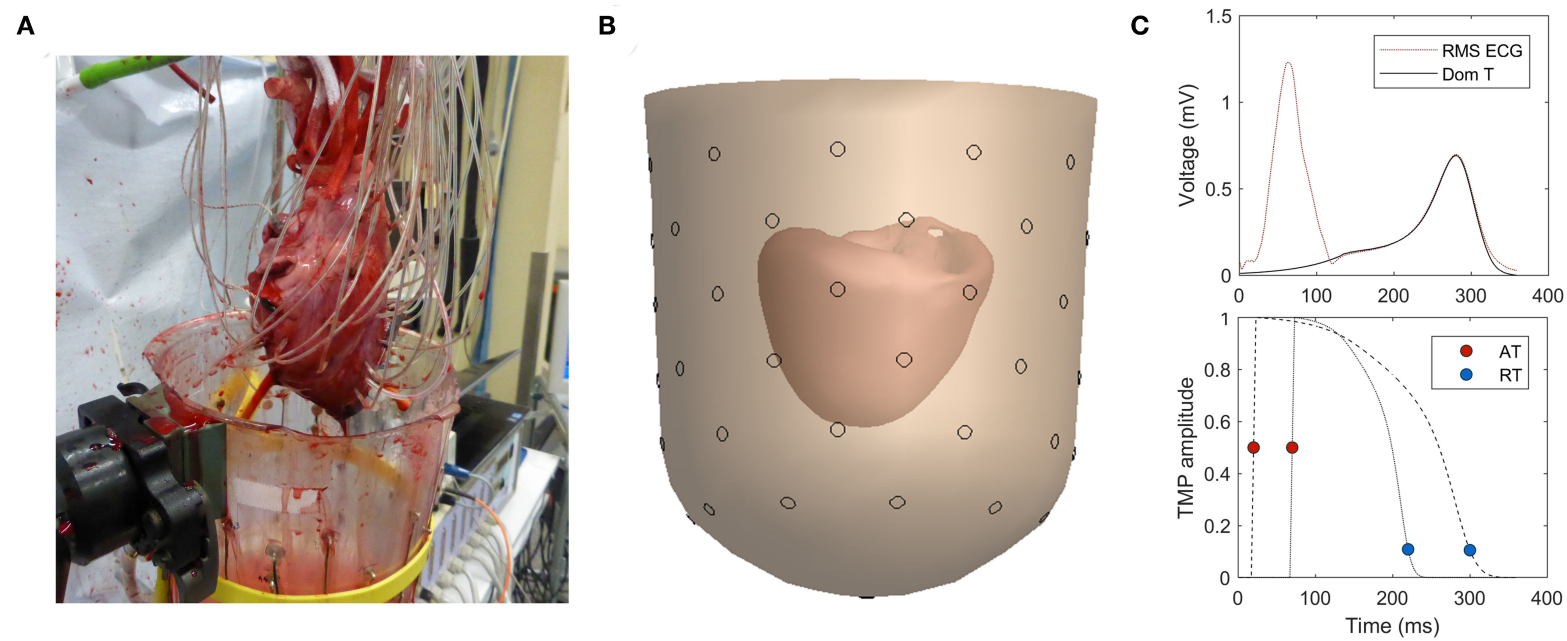
**(A)** Experimental setup with needles in myocardial wall. **(B)** 3D reconstruction of heart inside a fluid-filled container. **(C)** Dominant T-wave determined from T-wave (top) provides the template for the time course of the transmembrane potential (TMP) (bottom; example for two different heart nodes).

A total of 50 plunge needles (i.e., 3–4 electrodes each; interelectrode distance = 4 mm) were placed in all regions in the heart, allowing the recording of unipolar transmural electrograms. Pacing hooks for stimulation were placed on the left atrium and RV and LV epicardium. In addition, temperature sensors were placed on both the LAD- and aorta-perfused regions. Then, a cylindrical, fluid-filled container equipped with 61 surface electrodes was placed around the heart, allowing the recording of pseudo-body surface potential ECGs simultaneously with needle electrograms [BioSemi, The Netherlands; 2,048 Hz, bandwidth (−3dB) DC −400 Hz, 24-bit dynamic range, 122.07 nV least significant bit (LSB), total noise = 0.5 μV]. The apex of the heart was magnetically fixed to the bottom of the container to avoid swinging of the heart but allowing rotational and vertical movement (Figure 1A). A minimum time period of 30 min between needle insertion and recording was ensured to reduce the effects of injury current on repolarization as much as possible.

After the experiment, the geometry of the container with the heart and the needles inside the heart was detected using CT images from a Philips Brilliance 64, Best, The Netherlands (tube voltage: 120 kVp, tube charge: 495 mAs, voxel size: 0.33 × 0.33 × 0.33 mm, convolution kernel: D).

### Data Analysis

From the CT images, the geometry of the heart (i.e., epicardial and endocardial ventricular surfaces) and the container were reconstructed with GeomPeacs software (Peacs, The Netherlands) (Figure 1B). Needle positions were determined in three dimensions by tracking the needles in the images and using the known interelectrode distances. Electrodes with >5 mm distance of the reconstructed myocardial surface were excluded, reducing the data set to endocardial and epicardial electrode sites. Furthermore, electrodes with ST-elevation or flat T-waves were excluded. From the measured remaining unipolar electrograms, ATs were determined as the maximum downward slope of the QRS complex, and RTs were determined as the maximum upward slope of the T-wave (Haws and Lux, 1990; Coronel et al., 2006) with respect to the onset of QRS on the pseudo-ECG in atrial beats or stimulus artifact in ventricular paced beats. The electrophysiological analyses were performed using custom-made software based on MATLAB (Potse et al., 2002).

To determine the region that was separately perfused by the LAD, the following three aspects were investigated: (1) CT with contrast in LAD, (2) changing QT time and T-wave polarity on needle electrodes, and (3) photos from the heart where LAD was shortly blocked, giving a slightly blue color to its perfusion region. An estimate for the LAD perfusion area was delineated in the heart geometry by manually combining this information. The delineation of LAD was performed before the inversely reconstructed RT maps were available.

### ECG Imaging

The inverse computation was performed using the EDL method, as previously described in the study by van der Waal et al. (2020). A brief explanation is given in this study. A template for the time course of the TMP was constructed per heartbeat based on the dominant T-wave of the (pseudo-)body surface potentials (van Oosterom, 2003) (Figure 1C). For individual nodes at the myocardial surface, this template is shifted and stretched to match the AT and RT of that node. This TMP is used as source activity to compute the resulting potential at the electrode positions on the (pseudo-)body surface by using the lead field matrix.

The EDL method estimates AT and RT based on the body surface potentials, between which there is a nonlinear relation. Therefore, a nonlinear parameter estimation procedure is needed, which requires an initial estimate. The initial estimate for activation is provided by the fastest route algorithm; each node on the heart surface is iteratively considered as an initial focus, and the corresponding ATs on the heart surface is computed, assuming different propagation velocities along the myocardial surface (van Dam et al., 2009). The activation pattern whose corresponding body surface potentials best correlate with the measured body surface ECGs is chosen as the initial estimate. In the same way, the His-conducted beats up to five additional foci are added until there is no further significant improvement of the correlation. As an initial estimate for repolarization, a pattern that is inversely dependent on ATs is used for atrially paced beats, while for ventricularly paced beats, the activation estimate is used with a time delay, as explained in the study by van der Waal et al. (2020).

The EDL-based inverse entails the forward calculations of body surface potentials from assumed AT and RT. Starting from the initial estimates, the activation and repolarization patterns were iteratively optimized by minimizing the difference between the measured and estimated body surface potentials. The activation and repolarization patterns in the last step of the iterations are the patterns that produce the body surface potentials best matching the measured body surface potentials.

Since the inverse problem of ECG is ill-posed and therefore highly sensitive to measurement and modeling noise, regularization is needed to guard against unphysiological solutions. Similarly, in our previous study (van der Waal et al., 2020), we used second-order Tikhonov regularization: the surface Laplacian of the AT and RT was used as a penalty function in the optimization process, thus suppressing excessive irregularities in the activation and repolarization patterns (Huiskamp, 1991).

### Data Presentation

The correlation coefficients (CORs) and relative difference (RD) values between the measured and calculated pseudo-ECGs were determined. The RD was defined as the Frobenius norm of the difference between the simulated and reconstructed signals relative to the simulated data. The difference between repolarization timing for the measured and reconstructed patterns on electrode locations is presented as the mean ± SD of the absolute difference. The differences between groups were tested using Mann–Whitney *U* test, with *p*-values less than 0.05 considered statistically significant.

## Results

In the 4 explanted pig hearts, the following 16 beats were analyzed: atrially (*n* = 8), LV (*n* = 4), or RV (*n* = 3) paced beats, atrially paced beats with cold infusion (*n* = 4), and an atrially paced beat with pinacidil infusion. The number of needle electrodes included was 82.2 ± 16.3 (range = 59–115). A single beat was analyzed for each condition. In Supplemental Materials, we have shown that the results are similar for subsequent beats.

### Atrially Paced Beats

**Figure 2:**
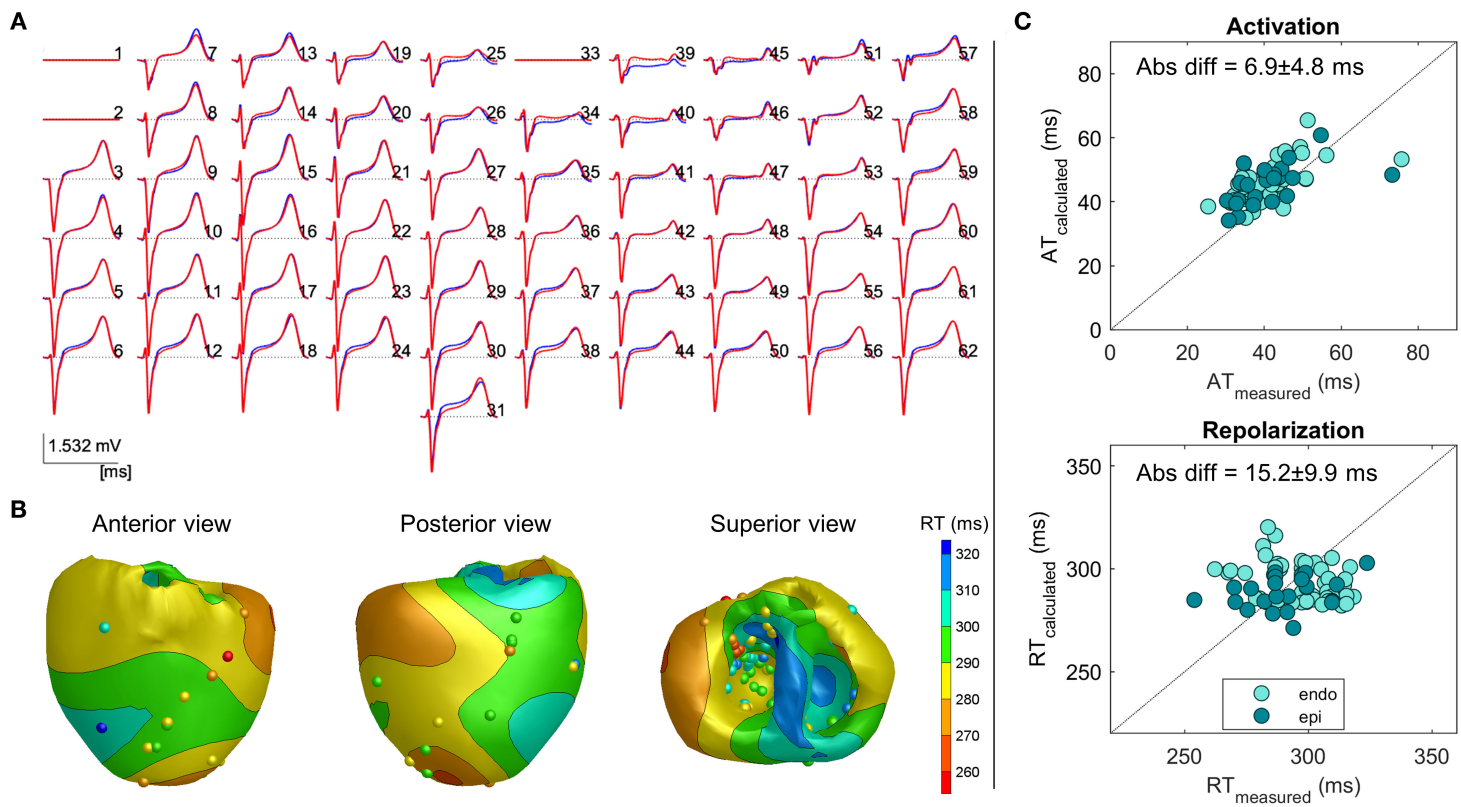
Example 1: Inverse reconstruction of heart 4, atrially paced beat. **(A)** Superimposed measured (blue) and reconstructed (red) body surface ECGs. Isoelectric lines represent bad recording electrodes (channels 1, 2, and 33). **(B)** Three views of the heart, with surface color indicating the inverse calculated repolarization pattern, spheres indicating electrode measurement sites, and color indicating the corresponding measured repolarization time (RT). **(C)** Measured vs. calculated activation time (AT; upper panel) and RT (lower panel) on electrode sites, with a distinction between the endocardial and epicardial sites.

Figure 2 shows an example of a reconstructed repolarization pattern (i.e., upper panel) in a slow atrially paced beat with the corresponding pseudo-ECGs in heart 4. There is good agreement between the reconstructed (red) and measured (blue) pseudo-ECGs (panel A), with a correlation of 0.99 and RD of 0.28. The mean absolute difference between the recorded and measured RTs was 15.2 ± 9.9 ms. The minimum and maximum RTs are very similar between the reconstructed and measured points (min: 268 vs. 254 ms, max: 321 vs. 324 ms, respectively). Scatterplots of measured vs. calculated ATs and RTs are shown on the right. The data points cluster around the line of identity. The epicardial and endocardial RT data show close correspondence in accuracy.

Table 1 enumerates the accumulated data for each of the individual hearts during each of the conditions. For the atrially paced beats of all hearts in the baseline condition, the overall difference between the calculated and measured RTs was 21.8 ± 19.6 ms (*n* = 8). Although some deviations occurred, especially in the minimum value of RT, the range of the reconstructed RT pattern was similar to that of the measured RT pattern (Table 1). This is likely related to the local ST-elevation caused by the insertion of the needles (refer to the “Discussion” section).

**Table 1:** Comparison of the parameters between the reconstructed and measured pseudo-ECGs and the repolarization times for different conditions per heart (H1 to H4).

	**Beat**	**COR ECG**	**RD ECG**	**Abs diff rep (ms)**	***N* electrodes**	**Range reconstructed (ms)**	**Range measured (ms)**
H1	*Atrial_s*	0.86	0.59	25.2 ± 21.4	59	138–263	183–250
	*Atrial_f*	0.85	0.58	28.3 ± 26.8	59	93–240	158–228
	*LV*	0.85	0.59	17.8 ± 11.9	71	192–294	181–276
	*Cold*	0.91	0.56	36.3 ± 23.6	68	188–318	188–313
H2	*Atrial_s*	0.91	0.46	21.8 ± 21.5	98	225–359	236–366
	*Atrial_f*	0.96	0.31	32.1 ± 23.0	102	235–307	175–313
	*LV*	0.98	0.25	24.5 ± 16.9	87	242–345	221–346
	*RV*	0.97	0.25	16.6 ± 10.0	91	254–323	235–331
	*Cold*	0.92	0.41	38.8 ± 33.1	115	260–411	212–427
	*Pina*	0.96	0.29	37.0 ± 25.3	111	245–333	217–339
H3	*Atrial_s*	0.93	0.39	14.0 ± 12.8	69	243–340	263–330
	*Atrial_f*	0.96	0.32	16.4 ± 12.8	71	218–303	202–289
	*LV*	0.96	0.32	21.9 ± 15.5	80	227–334	223–330
	*RV*	0.95	0.31	18.2 ± 14.0	79	228–353	220–335
	*Cold*	0.96	0.33	36.3 ± 25.5	75	258–414	215–407
H4	*Atrial_s*	0.99	0.28	15.2 ± 9.9	79	268–321	254–324
	*Atrial_f*	0.99	0.27	18.4 ± 16.1	93	230–276	175–289
	*LV*	0.99	0.21	14.2 ± 10.9	79	240–299	213–314
	*RV*	0.97	0.31	16.0 ± 12.9	63	189–300	230–310
	*Cold*	0.98	0.27	48.8 ± 22.8	94	291–371	197–443
Accumulated absolute error				25.7 ± 22.3	1643	93–411	158–443

*COR, correlation; RD, relative difference; Abs diff rep, the absolute difference in repolarization time (RT); Range, min-max RT; Atrial_s, slow atrially paced beat; Atrial_f, fast atrially paced beat; LV/RV, left/right ventricularly paced beat; Pina, pinacidil perfusion; Cold, cold perfusion*.

We then studied the repolarization differences induced by ventricular pacing associated with secondary T-wave changes.

### Ventricularly Paced Beats

**Figure 3:**
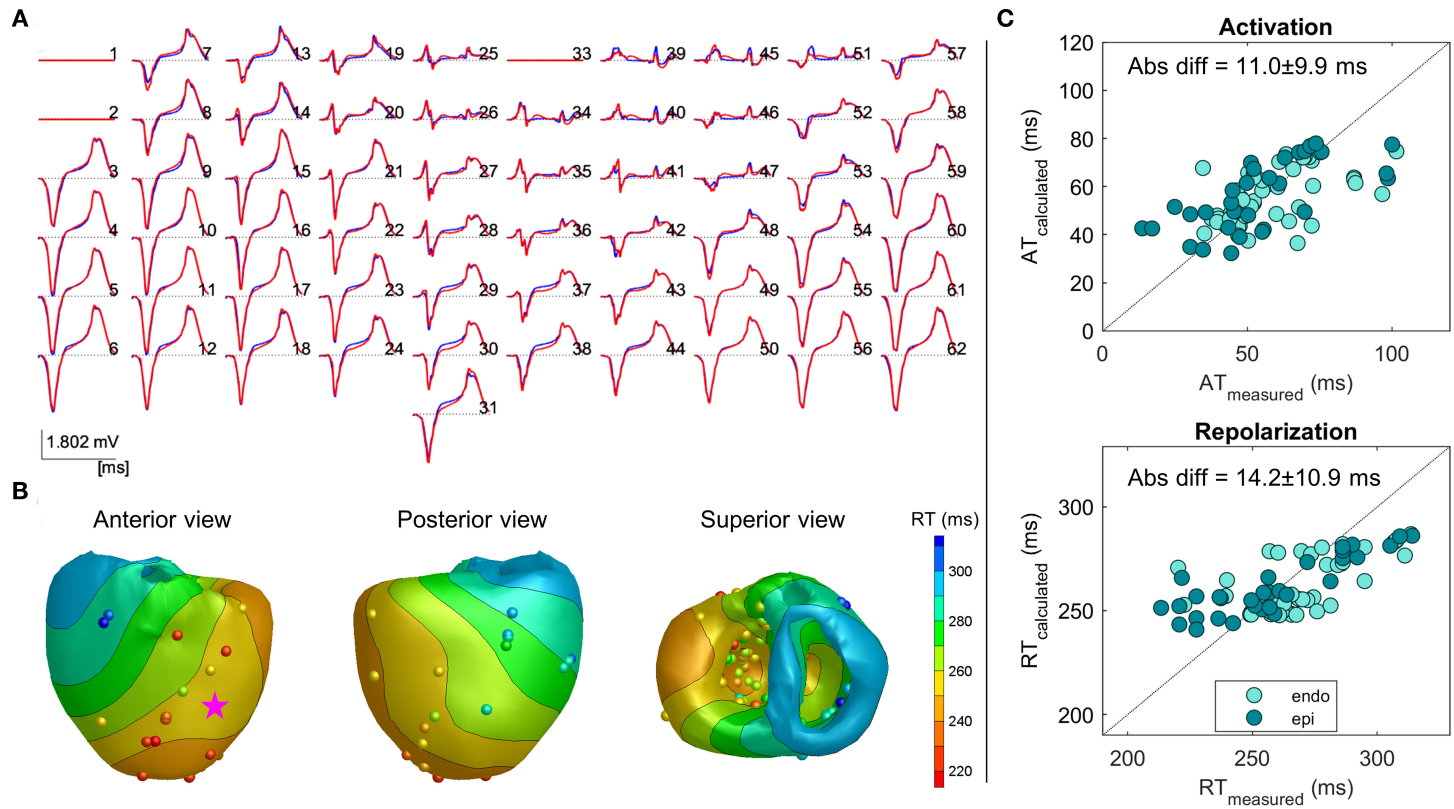
Example 2: Inverse reconstruction of heart 4, left ventricular (LV) paced beat (pink star indicating pacing location). The configuration is similar to that mentioned in Figure 2.

In Figure 3, an example of the same heart, as shown in Figure 2, with the reconstruction of an LV paced beat is shown. The reconstructed pseudo-ECG matched the measured ECG well, with a COR of 0.99 and RD of 0.21. Pacing from the ventricular site (star) led to a different repolarization pattern, as expected with the earliest repolarization occurring close to the pacing site (Figure 2B vs. Figure 3B). The absolute difference between the recorded and measured RTs was 14.2 ± 10.9 ms. Thus, also in the ventricularly paced beats, the calculated RTs are similarly accurate for both endocardium and epicardium.

Overall, as shown in Table 1, the inverse estimation of the ventricularly paced beats (LV and/or RV) of all the hearts performed similarly to that of the atrially paced beats (*p* = 0.6), with an overall mean difference of 18.6 ± 13.7 ms (*n* = 7) between the calculated and measured RTs. Also, the range of the calculated RTs was accurately represented.

### Dispersion of Repolarization

The dispersion of repolarization has been shown to be arrhythmogenic (Coronel et al., 2009). Therefore, we tested the inverse reconstruction in conditions with artificially increased localized RT gradient.

We increased RT gradients by regional cooling in all four hearts. The process involved decreasing the temperature locally by infusion of cold, oxygenating perfusion fluid in the LAD region of the heart, resulting in the lowest temperatures of 34.3°C, 30.6°C, 33.5°C, and 30.1°C, and was accompanied by a local increase in the measured RTs of 64, 107, 144, and 119 ms for hearts 1–4, respectively. The atrially paced beats were studied.

**Figure 4:**
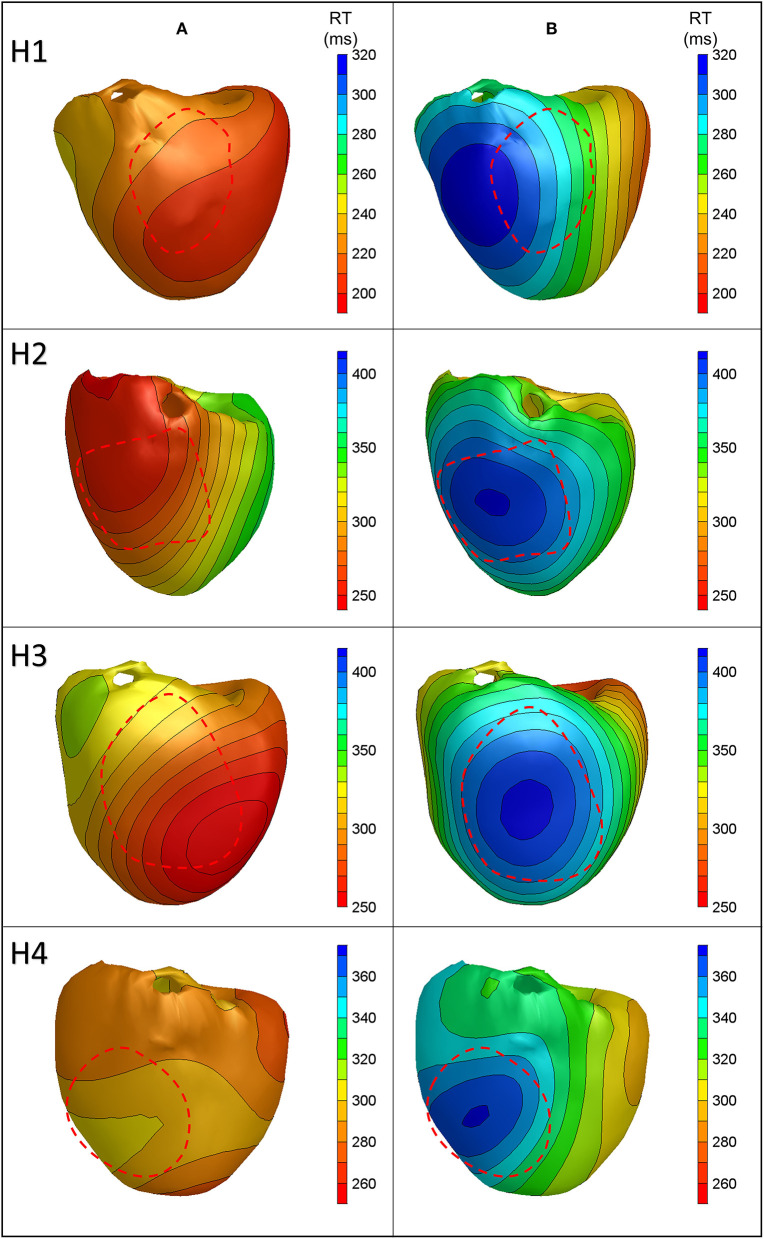
Anterior views of reconstructed repolarization times for atrially paced beats in all hearts with **(B)** and without **(A)** cold perfusion of the left anterior descending artery (LAD) region. The red dashed line indicates the estimated LAD perfusion area.

Figure 4 shows the anterior view of the reconstructed cold perfusion beat for all four hearts, with red lines indicating the estimated LAD perfusion area. Notably, the selectively perfused tissue is accurately represented in hearts 2 through 4, with the location of maximum reconstructed RT roughly in the middle of the estimated LAD-perfused region. In heart 1, the shape of the reconstructed pattern is similar, but the location is shifted to the left. This may have been caused by the postmortem deformation of the heart during CT (refer to the “Discussion” section). The distance between the location of maximum RT and the center of the LAD-perfused region is about 2.5 cm in this heart. The total absolute difference between the measured and calculated RTs for all cold-perfused atrially paced beats was greater than normal atrially perfused beats (40.4 ± 27.6 ms vs. 21.6 ± 19.7 ms, *p* < 0.01). This may be explained by the fact that the regularization of the inverse method smoothens the steep gradient in RT at the margin of the cold region.

**Figure 5:**
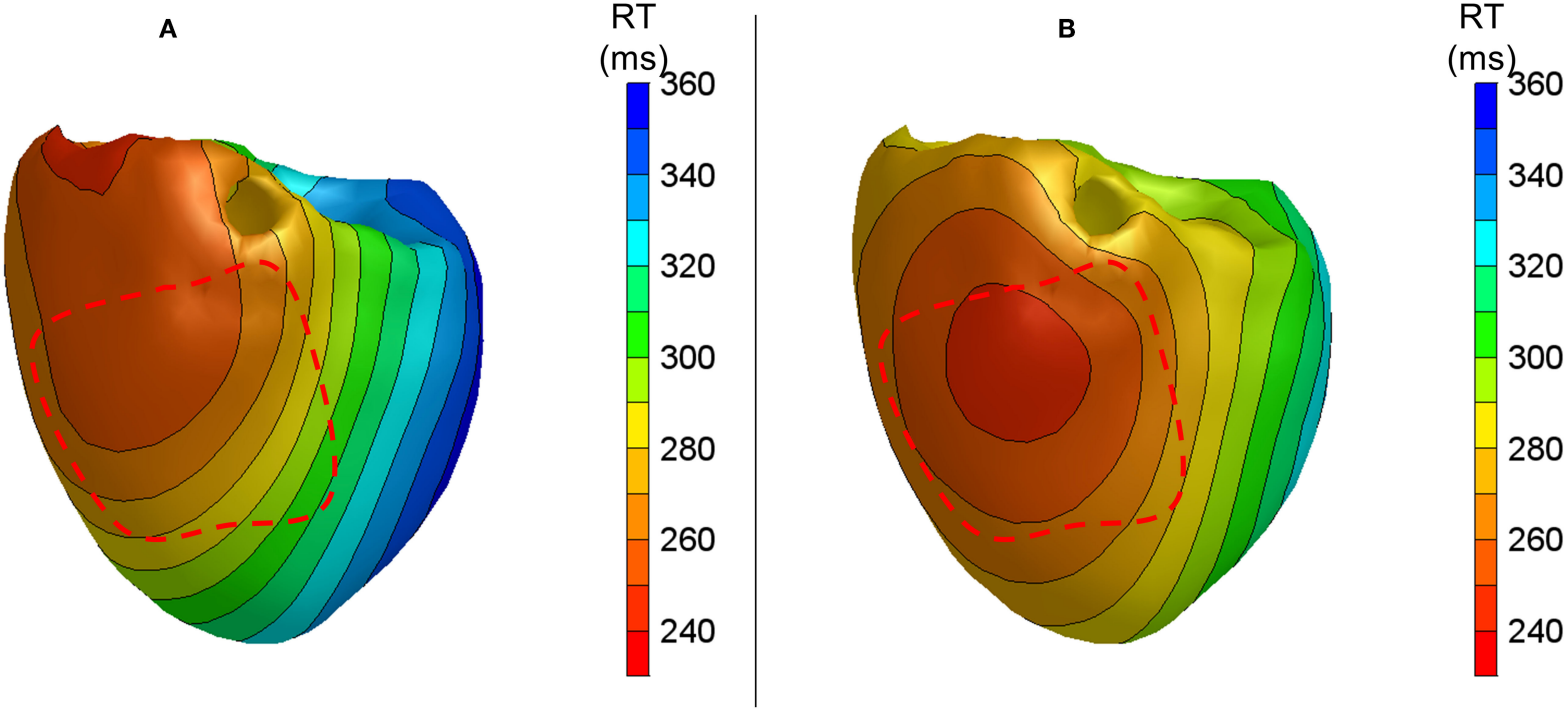
Anterior views of reconstructed repolarization times for atrially paced beats in heart 2 with **(B)** and without **(A)** local pinacidil perfusion of the LAD region. The red dashed line indicates the estimated LAD perfusion area.

After the temperature had reached the normal level, a local infusion of drugs was performed in the LAD region. In heart 2, pinacidil was infused, leading to the shortening of repolarization by about 40 ms. Figure 5 shows the repolarization patterns in an atrially paced beat with and without pinacidil infusion. It was noted that a shortening of the RT is observed in the LAD region, which is consistent with the area of infusion. The averaged difference between the measured and calculated RTs was 37.0 ± 25.3 ms (Table 1). Heart 1 did not show the repolarization shortening effect of pinacidil and was, therefore, excluded from the analysis of repolarization abnormalities.

For these four hearts, the localization of the regions with shortened (pinacidil) or prolonged (cold) RT had an error of a maximum of 2.5 cm. The absolute difference between the measured and calculated RTs is higher for the beats during increased and decreased RTs than for the baseline atrially or ventricularly paced beats (overall mean = 39.7 vs. 18.7 ms, *p* < 0.01). This is also related to a larger difference between minimum and maximum for the reconstructed and measured RTs. For normal atrially and ventricularly paced beats, the difference between the minimum reconstructed and measured RTs is 20.1 ± 13.1 ms and that between the maximum reconstructed and measured RTs is 9.7 ± 5.8 ms. For the beats with increased RT gradients, it is 42.6 ± 34.3 ms for the minimum and 21.2 ± 28.7 ms for the maximum difference between the reconstructed and measured RTs (all *p* < 0.01).

**Figure 6:**
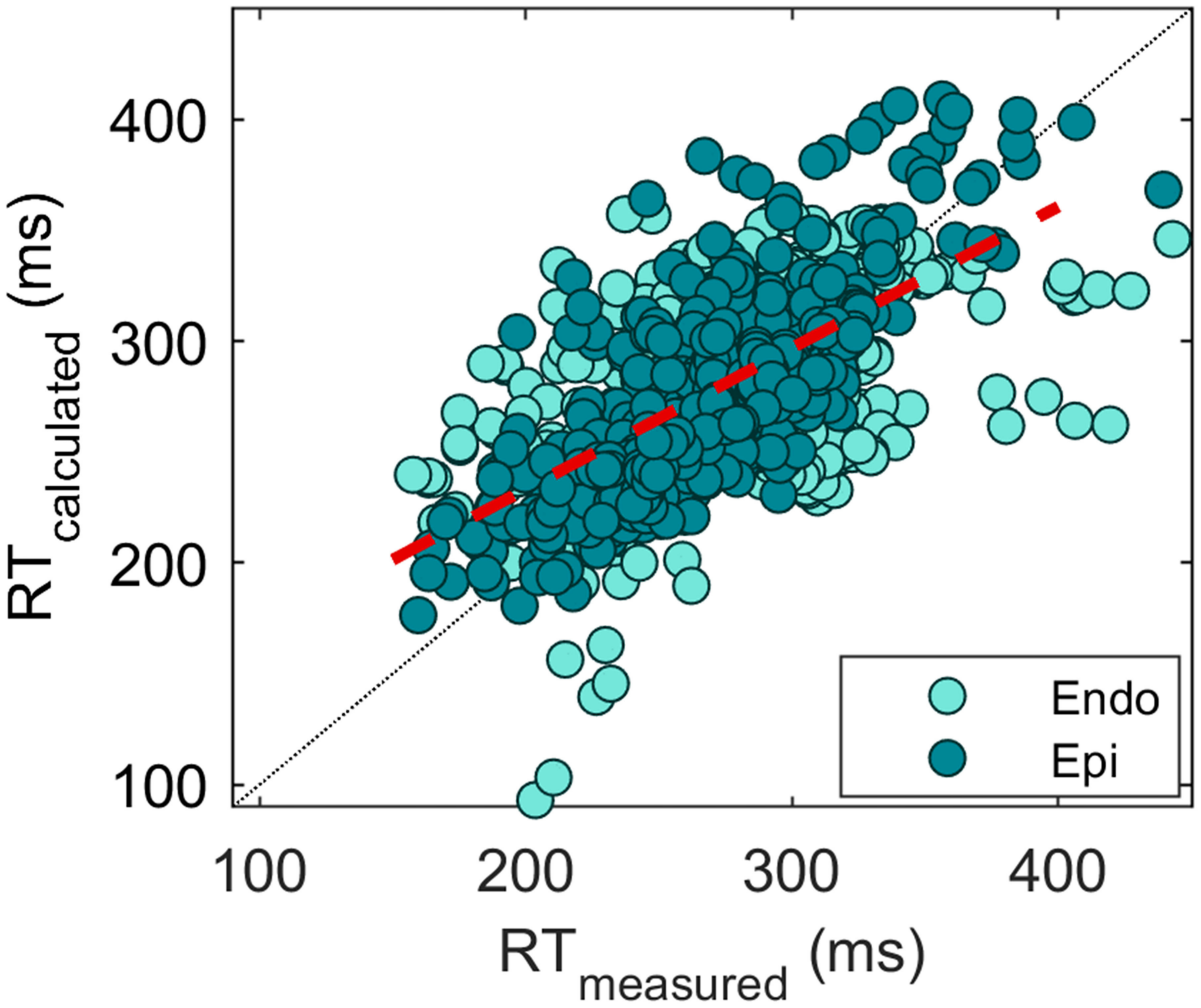
Scatterplot of measured vs. reconstructed RTs (*n* = 1,643). Each point indicates an electrode location in the heart. A distinction between the endocardial (turquoise) and epicardial (green) measurement sites is made. The overall correlation of 0.63 is depicted by the red dashed line.

Overall, 1,643 measured RTs were compared with the reconstructed RTs in different hearts and conditions. This resulted in an absolute error of 25.7 ± 22.3 with a COR of 0.63 (Figure 6). The error is slightly higher for endocardial validation points (27.8 ± 23.1 with the correlation of 0.53, *n* = 1,100) than for epicardial validation points (21.4 ± 20.0 with the correlation of 0.76, *n* = 543), *p* < 0.01. This may be related to the endocardium being more susceptible to errors in geometry determination due to the postmortem contracture of the heart (refer to the “Discussion” section).

## Discussion

In this study, we demonstrated that the EDL-based inverse methods allow the adequate reconstruction of RTs and repolarization patterns on the endocardium and epicardium of a heart in an *ex vivo* setting with an accuracy of about 25 ms. We showed that this inverse method detects the regions of prolonged and shortened repolarization, with a correct localization of the region of prolonged repolarization in 3 out of 4 hearts (with a localization error of ~2.5 cm in the other heart).

The error of about 20 ms for regular atrially and ventricularly paced beats between the measured and reconstructed RTs is slightly higher than the error that we found in our previous *in silico* validation study. In that study, we found validation errors ranging from 12 to 19 ms. However, in that study, the whole repolarization pattern (± 1,500 nodes) was taken into account in addition to the selected electrode sites (van der Waal et al., 2020). Given the perfect conditions that can occur only during an *in silico* study, the increased error in this study is to be expected. Moreover, an error of about 20 ms is physiologically similar to the activation errors (~13 ms in this study), given that the repolarization process occurs gradually and that the duration of the *T*-wave was 1.5–3.5 times longer than the QRS duration. These activation errors are comparable to the previous *in vivo* validation results (Oosterhoff et al., 2016).

No other validation studies regarding RTs with the EDL model have been performed. However, it is possible to compare our results to the validation results achieved at the epicardium with other inverse modeling approaches. The EP method was applied in a study by Zhang et al. on patients undergoing open-heart surgery (Zhang et al., 2017). They described that 78% of the 240 compared epicardial electrograms had a difference of less than 10 ms between the reconstructed and measured activation recovery intervals (ARIs). Cluitmans et al. also by using the EP method, reported a pooled correlation between the measured and calculated RTs of 0.73, which was measured in 4 canine hearts at 103 electrode positions (Cluitmans et al., 2017). Pooling our data of normal atrially and ventricularly paced beats from different hearts results in a correlation of 0.74 (*n* = 1,180 electrodes over 15 beats), while the inclusion of the beats with increased RT gradient results in a correlation of 0.63 (*n* = 1,643 electrodes over 20 beats).

A potential cause for errors in the inverse estimation of the RTs in our study is ST-elevation caused by the insertion of the needle electrodes. In some hearts, this ST-elevation was reflected on the body surface, causing the inverse method to try to resolve the ST-elevation by adjusting the local calculated RTs and ATs. This is not likely to yield an optimal solution (because it is known that ST-elevation is caused primarily by a change in resting membrane potential (Janse and Wit, 1989), which is not incorporated in the current implementation of the EDL model).

In contrast to the initial estimation of the ATs, the initial estimation of the RTs is not based on the recorded ECG but on the general assumptions of the order of repolarization in relation to the ATs (i.e., inverse to the activation sequence in atrially paced beats). Since the relationship between activation and repolarization is not so straightforward (Franz et al., 1987; Opthof et al., 2017), this approach may lead to a suboptimal initial estimate. Providing the EDL method with a better initial estimate for RTs will reduce the computational time (by decreasing the number of necessary optimization iterations) but, more importantly, will improve the likelihood of finding the correct repolarization pattern. Therefore, we plan to investigate an initial estimate for repolarization that also uses the recorded ECG (T-wave) in future studies.

### Dispersion of Repolarization

The dispersion of repolarization can lead to the existence of a “border” between regions with long vs. short RT (Rivaud et al., 2021). This gradient between two adjacent regions has the potential to create unidirectional block and reentry. Some dispersion of repolarization (based on the regional expression differences) is normal, but this may be amplified by the genetic and pharmacological factors (Opthof et al., 2017). Patients with the long-QT syndrome have increased dispersion of repolarization (Lubinski et al., 1998; Milberg et al., 2005), which may be aggravated by beta-adrenergic stimulation (Shimizu and Antzelevitch, 2000). In addition, the dispersion of repolarization might also explain the cases that were previously classified as idiopathic VF (Yazaki et al., 2011).

In this study, we have found that even though the absolute RT error was somewhat higher in the cold-perfused beats, the reconstructed repolarization pattern did accurately identify a region of delayed repolarization representing the cold perfusion region. This is similar to the errors in the localization of the pacing site in activation mapping (Oosterhoff et al., 2016; Cluitmans et al., 2017). This suggests that the resolution is high enough to guide invasive assessment to help improve outcome, although *in vivo* clinical validation is warranted. On the other hand, one should be aware that the accuracy of the inverse methods is not high enough to detect RT (or AT) changes on a millimeter-scale resolution, as some of the maps may suggest. This is especially relevant for the detection (using the EP method) of steep repolarization gradients that may be artifactual (Duchateau et al., 2019).

A study in a canine heart described the results of reconstruction using the EP model in the presence of local warming and cooling (thereby also creating gradients), showing a correlation between the measured and reconstructed ARI patterns of 0.75 with an average difference of 2 ms (Ghanem et al., 2001). The correlation for repolarization in our study was lower (0.64) with a larger average difference (25 ms). An important difference in methodology is that, in the study by Ghanem et al. (2001), the body surface ECG used to reconstruct the pattern was not recorded but calculated from the recorded EPs, which may have led to circular reasoning. Even though some perturbations were added (50 μV signal noise and 1 mm shift in body surface node location), this could lead to model-to-model bias. A more recent *ex vivo* pig study by Bear et al. (2021) created repolarization abnormalities by locally infusing dofetilide (causing RT prolongation) and pinacidil. The authors reported correlations of 0.73 for baseline and drug situations with a mean absolute error of 25 ms in RT. Following the same methods but with the EDL method that also includes the calculations of the endocardial surface, this study shows that the mean absolute error in all cases is very similar to 25 ms.

An inherent property of the methodology is the necessity for regularization, which guards against unphysiological solutions by suppressing excessive irregularities in the activation and repolarization patterns. However, in cases of sharp repolarization gradients, this leads to the smoothing of the gradient border. This can explain why we found absolute RT errors to be higher in the reconstructed beats with artificial RT gradients, especially in the minimum and maximum RTs. The effect of spatial smoothing is also described by Ghanem et al. using the EP method (Ghanem et al., 2001). The authors failed to identify the local minimum associated with LV cooling, due to a limitation in the spatial resolution of the approach as a result of regularization. In contrast, a study by Duchateau et al. using the EP method on patients documented artifactual steep activation and repolarization gradients (Duchateau et al., 2019). This difference between these two studies might be explained by the different regularization methods used.

### Clinical Relevance

The noninvasive detection of repolarization abnormalities potentially allows a better risk stratification for patients with electrical disease, such as long QT, early repolarization syndrome, and idiopathic VF. In addition, it allows the evaluation of the risk of these patients without the necessity of invasive diagnostic methods and the better selection of patients who may benefit from further invasive treatment. The correct localization of the region of late repolarization can also guide the physician to a region of interest in invasive electrophysiological studies, more than what can be achieved with the conventional 12-lead ECG T-wave analysis (even with a minor displacement of this region). The EDL method additionally helps to determine a primary endocardial or epicardial approach for invasive diagnostic studies.

### Methodological Considerations

Regional infusion of cold fluid in the LAD region resulted in RT differences between the normal and late repolarizing regions of up to 144 ms, with a relatively large region of prolonged repolarization. In clinical cases, RT gradients are likely to be more subtle. However, the exact relationship between the amount of repolarization dispersion and susceptibility to arrhythmias is not fully understood (and may also be dependent on other factors) (Killeen and Sabir, 2007; Rivaud et al., 2021).

In the current measurement setup, the heart is placed in an electrode-equipped container filled with conducting fluid. This does not reflect the placement of the heart in the torso. In the container, the distance between the heart and the electrodes is less, and other tissues (e.g., lungs and fat) in between that influence the measured signal on the body surface, are absent. The presence of lung and other tissues may reduce the accuracy of the inverse method in an *in vivo* setting. A source of inaccuracy in this study may be that the container with heart and needles was placed in the CT after performing the experiment. After the termination of sustaining Langendorff perfusion, the myocardium develops contracture, leading to an alternative heart morphology. This may have influenced the correctness of the heart geometry (possibly more on the endocardium). This may also have played a role in heart 1 in which a localization error of 2.5 cm was observed. In the clinical situation, the expected localization error, therefore, is less.

We compared reconstructed RT with RT that was determined from needle electrodes in the myocardium. As a result, we compared RT calculated on the myocardial surface with RT that was not exactly on the surface. We tried reducing this influence by excluding the measurement sites of >5 mm distance of the reconstructed myocardium. This led to a reduced amount and uneven distribution of measurement sites and denied us the possibility of extrapolating a full repolarization pattern from the measured RTs.

## Conclusion

We have validated the reconstruction of the endocardial and epicardial repolarization patterns by using the EDL method in isolated perfusion pig hearts under normal conditions (i.e., atrial pacing), secondary T-wave changes (i.e., ventricular pacing), and regional infusion of action potential prolonging or shortening drugs. A repolarization error of about 20–25 ms was observed for both endocardial and epicardial recoding sites, corresponding to other inverse techniques and also in line with the inaccuracy of AT detection. The technique potentially allows the early detection of patients with large repolarization gradients and opens new strategies for primary and secondary prevention. The noninvasive mapping of repolarization, therefore, can help to select patients who are in need of anti-arrhythmic drug therapy or triaging patients who require further (invasive) assessment.

## Data Availability Statement

The raw data supporting the conclusions of this article will be made available by the authors, without undue reservation.

## Ethics Statement

The animal study was reviewed and approved by Animal Research Institute AMC/Animal experiment committee AMC Academic Medical Center Amsterdam.

## Author Contributions

JW, VM, TO, and RC conceived the design of the study, with the development of the EDL technique by TO. VM, CB, GS, and RC prepared and performed the experiments, with GS focusing on the imaging part. JW performed the analysis, analyzed the results, and wrote the manuscript, with VM, TO, and RC involved in supervision and contributed to the final manuscript. All authors contributed to the revision of the manuscript, read, and approved the submitted version.

## Funding

This study was supported by the Technology Foundation STW (Grant No. 10959), the Leducq Foundation (RHYTHM transatlantic network, Grant No. 16CVD02), and ZonMw (GALANT, project number 116004202).

## Conflict of Interest

The authors declare that the research was conducted in the absence of any commercial or financial relationships that could be construed as a potential conflict of interest.

## Publisher's Note

All claims expressed in this article are solely those of the authors and do not necessarily represent those of their affiliated organizations, or those of the publisher, the editors and the reviewers. Any product that may be evaluated in this article, or claim that may be made by its manufacturer, is not guaranteed or endorsed by the publisher.
