# Supplementary material for: Ex vivo Validation of Noninvasive Epicardial and Endocardial Repolarization Mapping
Source: Front Physiol. 2021 Oct 22;12:737609. doi: 10.3389/fphys.2021.737609 (PMC8569864; doi:10.3389/fphys.2021.737609)
Supplement: Supplementary file 1 [file Data_Sheet_1.PDF]

Supplementary Material

1 Supplementary Results

Reproducibility of subsequent beats

We tested the reproducibility of the ECGI reconstruction for consecutive, unique beats, to demonstrate the reliability of the method for subsequent beats. We used an LV paced beat from a single heart (heart 4), and selected three subsequent beats. Resulting repolarization patterns are shown in Supplemental Figure 1, with only very minor differences between repolarization patterns. Supplemental Figure 2 shows the corresponding scatterplots for activation and repolarization, and numerical results are summarized in Supplemental Table 1. We conclude that ECGI produces repolarization patterns that are reproducible for subsequent beats.

1.1 Supplementary Figures

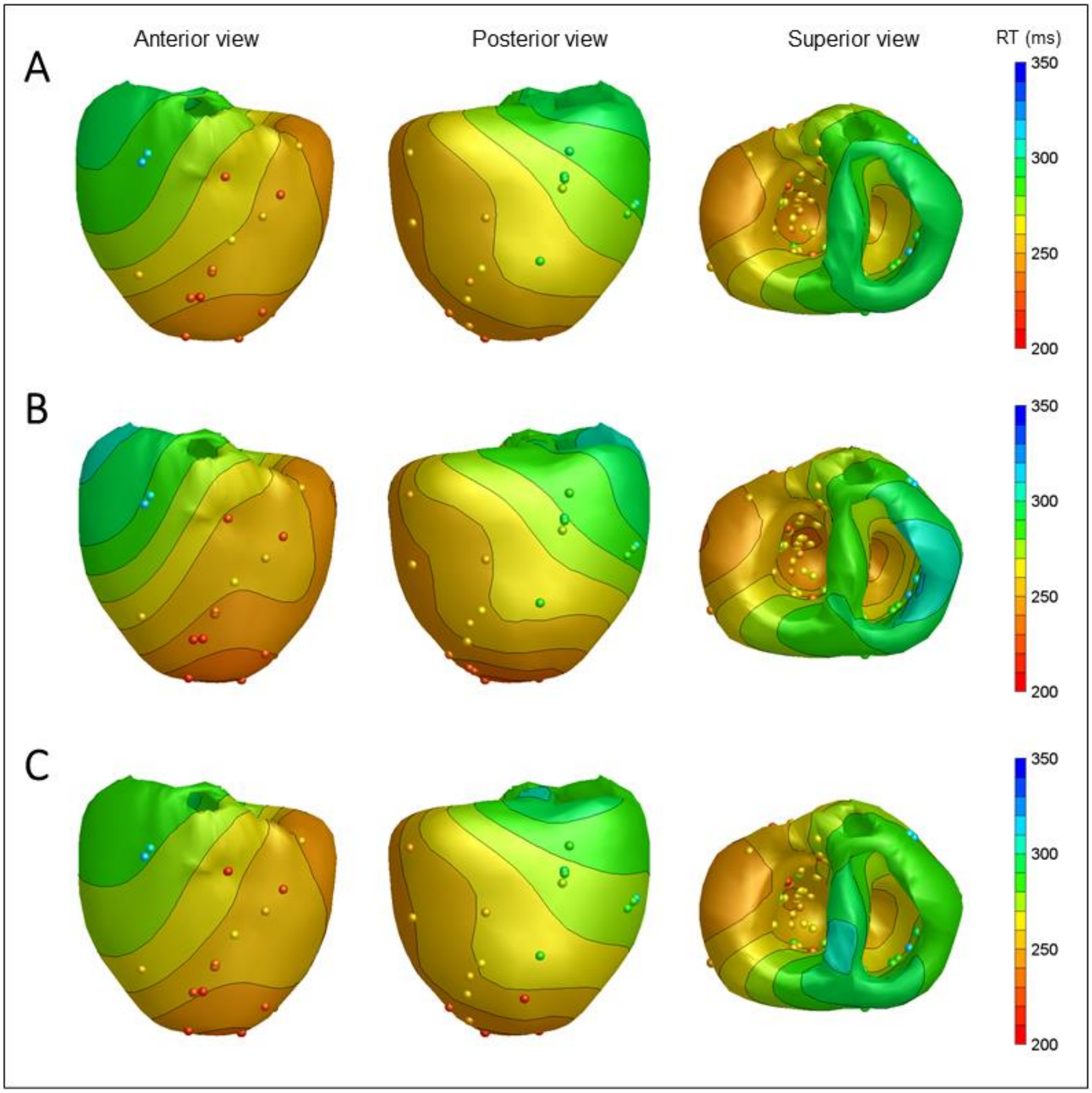

Supplementary Figure 1. Repolarization patterns of heart 4, LV paced beat. Surface color indicating the inverse calculated repolarization pattern and spheres indicating electrode measurement sites with color indicating the corresponding measured RT. Panel A shows the repolarization pattern included in the main text, panel B and C show results for subsequent beats.

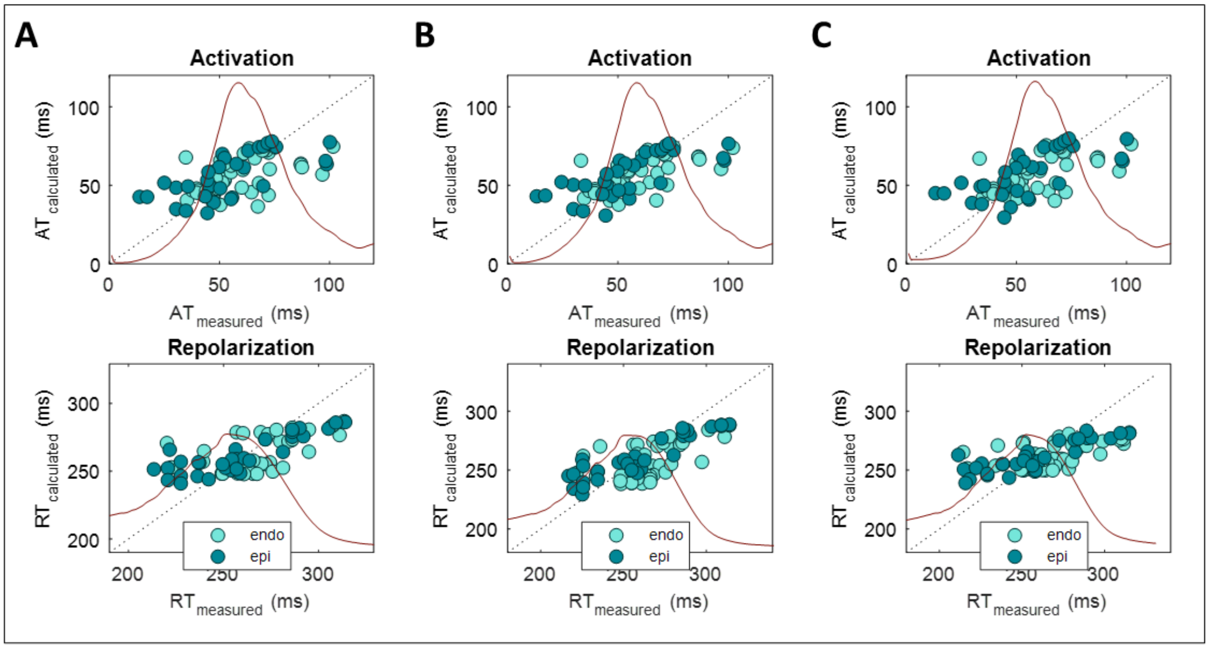

Supplemental Figure 2: Measured versus calculated AT (upper panel) and RT (lower panel) of subsequent repolarizations corresponding to the maps in supplemental figure 2 (A,B,C) on electrode sites. Endocardial and epicardial sites are indicated by differently colored dots. For reference, the root-mean-square of the corresponding QRS complex and T-wave are plotted within the figure.

Supplemental Table 1: Comparison parameters between reconstructed and measured pseudo-ECGs (Cor=correlation, Rd=relative difference) and repolarization times (Abs diff rep=absolute difference in RT, Range= min-max RT) for three subsequent LV paced beats.

	**Beat**	**Cor ECG**	**Rd ECG**	**Abs diff rep (ms)**	**N electrodes**	**Range reconstructed (ms)**	**Range measured (ms)**
**H4**	*LV_1*	0.99	0.21	14.2±10.9	79	240 - 299	213 - 314
	LV_2	0.99	0.21	14.4+-9.6	79	227 – 313	216 – 314
	LV_3	0.99	0.21	15.5+-12.3	79	238 – 302	211 – 315
